# Inhibition of apoptosis-regulatory protein Siva-1 reverses multidrug resistance in gastric cancer by targeting PCBP1

**DOI:** 10.32604/or.2022.027301

**Published:** 2023-02-09

**Authors:** FANBIAO KONG, KUN WU, LIMING PANG, YULIANG HUANG, LEI LI, JING XU, FEITONG LI, YAN QING, ZHONGYU WANG, XIURONG HUANG, SHENG XU, XIAOGANG ZHONG, ZHOU ZHU, XIAOTONG WANG, JIANRONG YANG

**Affiliations:** 1 Department of General Surgery, The First Affiliated Hospital of Jinan University, Guangzhou, 510630, China; 2 Department of Colorectal and Anal Surgery, The People’s Hospital of Guangxi Zhuang Autonomous Region & Institute of Minimally Invasive Technology and Applications, Guangxi Academy of Medical Sciences, Nanning, 530021, China; 3 Departments of Gastrointestinal, Hernia and Enterofistula Surgery, People’s Hospital of Guangxi Zhuang Autonomous Region & Institute of Minimally Invasive Technology and Applications, Guangxi Academy of Medical Sciences, Nanning, 530021, China; 4 Departments of Hepatobiliary and Gastrointestinal Surgery, Minzu Hospital of Guangxi Autonomous Region, Nanning, 530001, China; 5 Department of Hepatobiliary, Pancreas and Spleen Surgery, The People’s Hospital of Guangxi Zhuang Autonomous Region & Institute of Minimally Invasive Technology and Applications, Guangxi Academy of Medical Sciences, Nanning, 530021, China

**Keywords:** Multidrug resistance, Gastric cancer, Siva-1, PCBP1

## Abstract

**Introduction:**

Siva-1, as a pro-apoptotic protein, has been shown to induce extensive apoptosis in a number of different cell lines. In our previous study, we showed that overexpressed Siva-1 decreased the apoptosis of gastric cancer cells. So, we believe that it can also work as an anti-apoptotic protein. The present study aimed to determine the specific role of Siva-1 in anticancer drug resistance in gastric cancer in vivo and in vitro and preliminarily reveal the mechanism.

**Materials and Methods:**

A vincristine-resistant MKN-28/VCR gastric cancer cell line with stably downregulated Siva-1 was established. The effect of Siva-1 downregulation on chemotherapeutic drug resistance was assessed by measuring the IC50 and pump rate of doxorubicin. Proliferation, apoptosis of cells, and cell cycle were detected via colony formation assay and flow cytometry, respectively. Additionally, migration and invasion of cells was detected via wound healing and transwell assays. Moreover, we determined *in vivo* effects of LV-Siva-1-RNAi on tumor size, and apoptotic cells in tumor tissues were detected using TUNEL and hematoxylin and eosin staining.

**Results:**

Siva-1 downregulation reduced the pump rate of doxorubicin and enhanced the response to drug treatment. Siva-1 negatively regulated proliferation and promoted apoptosis of cells by potentiality G2-M phase arresting. Inhibition of Siva-1 expression in MKN-28/VCR cells significantly weakened wound healing ability and decreased invasion ability. Poly(C)-binding protein 1 (PCBP1) was identified as a Siva-1-interacting protein in yeast two-hybrid screening. Semiquantitative RT-PCR and western blotting revealed that Siva-1 downregulation could inhibit expression of PCBP1, Akt, and NF-κB and eventually decrease the expression of MDR1 and MRP1.

**Conclusion:**

he current study demonstrated that the downregulation of Siva-1, which functions as a regulator of MDR1 and MRP1 gene expression in gastric cancer cells by inhibiting PCBP1/Akt/NF-κB signaling pathway expression, enhanced the sensitivity of gastric cancer cells to certain chemotherapies.

## Introduction

Cancer is responsible for nearly 1 in 6 deaths worldwide [1]. Breast, cervical, lung, thyroid, and colorectal cancers are the most common types of cancer in women, whereas prostate, lung, colorectal, liver, and stomach cancers are the most common among men [2]. Even though there are different methods of cancer treatment including radiation therapy, surgery, immunotherapy, endocrine therapy, and gene therapy, chemotherapy remains the most common method of treating cancer [3]. However, chemotherapy has limited efficacy due to the development of multidrug resistance (MDR). MDR leads to chemotherapy failure typically associated with a decrease in the drug concentration inside cancer cells, commonly due to MDR1 and MRP1 overexpression, which limits the efficacy of chemotherapeutic drugs [4,5].

Siva-1 is a typical apoptotic protein commonly activated by the p53 tumor suppressor protein and should participate as a barrier against the development of cancer [6]. Many studies have demonstrated an anti-apoptotic role of Siva-1 in several malignant tumors [7,8]. Our previous data demonstrated that the percentage of apoptotic cells decreased following overexpression of Siva-1. Additionally, overexpression of Siva-1 functions as a regulator of MDR1 and MRP1 gene expression via promotion of NF-κB expression [5]. Siva-1 plays an important role in regulating the sensitivity of gastric cancer cells to certain chemotherapies. The aim of this research was to investigate the reversal effect of Siva-1 on MDR in human gastric cancer.

## Materials and Methods

### Reagents

Trypsin (T4549), streptomycin (85886), penicillin (V900929), and vincristine (VCR) were obtained from Sigma-Aldrich (Shanghai, China). MKN-28/VCR cells were cultured in RPMI-1640 medium (Invitrogen, Thermo Fisher Scientific, Waltham, MA, USA) with fetal bovine serum (B8687; Sigma-Aldrich,). Siva-1 (#12532), Poly(C)-binding protein 1 (PCBP1; #8534), Akt (#9272S), NF-κB (#8242), MRP1 (#72202), MDR1 (#13342), and GAPDH (#5174) antibodies were purchased from Cell Signaling Technology, Inc., USA. MTT assay kits (ab211091) and Akt pathway specific inhibitor LY 294002 (ab120243) were purchased from ABCAM (Shanghai, China). Reverse transcriptase-polymerase chain reaction (RT-PCR) kits were purchased from Thermo Fisher Scientific.

### Cell culture

MKN-28/VCR gastric cancer cells, which were obtained from the Experimental Center of the People’s Hospital of Guangxi Zhuang Autonomous Region, were cultured in RPMI-1640 supplemented with 10% fetal bovine serum (Sijiqing Biotec Co., Ltd., Hangzhou, China) and antibiotics (100 U/ml penicillin and 100 mg/ml streptomycin) and then incubated at 37°C in a humidified 5% CO_2_ atmosphere. MKN-28/VCR cells were maintained in culture medium supplemented with 0.6 μg/ml VCR to maintain the drug-resistance phenotype.

### Gene transfection

Recombinant lentiviral vector for Siva-1 gene (LV-Siva-1-RNAi) and null vector (Lv-NC), purchased from the Shanghai Cancer Institute (Shanghai, China), were used to construct the Siva-1-downregulation lentivirus. The methods of LV-Siva-1-RNAi and transfection of MKN-28/VCR gastric cancer cells with LV-Siva-1-RNAi have been previously described [5]. MKN-28/VCR cells were seeded in 6-well plates with antibiotic-free medium. After incubation for 24 h, cells were infected with recombinant lentivirus at a multiplicity of infection (MOI) value of 12 PFU per cell (MOI = 12). Transfected cells were cultured in G418 (600 mg/ml; Invitrogen; Thermo Fisher Scientific) for 4 weeks, after which stably downregulated cell lines were generated. The different MKN-28/VCR wells were then divided into three groups: an MKN-28/VCR-shRNA-Siva-1 group, MKN-28/VCR-shRNA-NC group, and MKN-28/VCR group.

### Measurement of cell viability

The MTT assay is a widely used approach for measuring cell viability. First, MKN-28/VCR cells (5 × 10^4^ cells/ml) were seeded into 96-well culture plates (100 μl/well) and exposed to VCR (1.8 μg/ml). Next, MTT solution (0.1 mg) was added to each well. After 4-h incubation at 37°C, 150 μL DMSO was added to each well to dissolve all precipitate, and the culture plate was placed on a rocker for 10 min at low speed. Finally, the optical density (OD) value was measured using a microplate reader (PR 3100 TSC; Bio-Rad Laboratories, Inc., Hercules, California, USA) at 450 nm. Relative drug resistance was analyzed and compared using IC50 values.

### Measurement of pump rate of doxorubicin by flow cytometry

The pump rate of doxorubicin was determined by measuring the fluorescence intensity of doxorubicin in cells using flow cytometry. MKN-28/VCR cells were seeded into 6-well plates, after which doxorubicin was added to each well to a final concentration of 4 mg/ml. Samples were then incubated at 37°C for 30 min. Doxorubicin levels were determined using an EPICS XL-MCL flow cytometry system (Beckman Coulter) with an excitation wavelength of 488 nm and an emission wavelength of 575 nm. All cells were subsequently washed twice with fresh culture medium and incubated at 37°C for 1 h to detect the retained doxorubicin. Subtraction of the fluorescent retained from the total fluorescence was the fluorescent index of doxorubicin. The procedure was performed in triplicate and an average value was obtained to calculate the pump rate of doxorubicin using the following formula: releasing index = (accumulation value−retention value)/accumulation value. The data were analyzed using MultiCycle software for Windows (version 3.0; Phoenix Flow Systems, San Diego, CA, USA).

### Effect of Siva-1 silencing on cell cycle control

MKN-28/VCR-shRNA-Siva-1 cells (1 × 10^6^) were repeatedly washed with ice-cold PBS, detached with trypsinization, and fixed in cold 70% ethanol (700 μL) at 4°C for 30 min. Subsequently, the cells were washed twice with ice-cold PBS in cold centrifugation for 10 min (4000 rpm). The resultant cell pellet was resuspended and incubated in a solution containing 50 mg/ml propidium iodide, 0.2 mg/ml RNase, and 0.1% Triton X-100 at 37°C for 30 min. The cells were analyzed with flow cytometry using an EPICS XL-MCL FACScan (BC, Mountain View, CA, USA). The data were analyzed with MultiCycle for Windows (Phoenix Flow Systems).

### Quantification of apoptosis via flow cytometry

To detect apoptosis om MKN-28/VCR-shRNA-Siva-1 cells, cultured cells in logarithmic growth phase were detached with 0.25% trypsin. Cells (1 × 10^6^) were washed repeatedly with ice-cold PBS and fixed in cold 70% ethanol at 4°C for 30 min. The cell pellet was incubated in a solution containing 10 μl/ml Annexin V-FITC, and 10 μl/ml 7-AAD at 37°C for 15 min in the dark, as recommended by the manufacturer. The cells were then analyzed by flow cytometry using an EPICS XL-MCL FACScan. The data were also analyzed with MultiCycle.

### Colony formation assay

A colony-forming unit assay was used to detect the proliferation and differentiation of human gastric cancer MKN-28/VCR-shRNA-Siva-1 cells. Cells were seeded in 6-well plates and incubated in the presence of VCR (0.6 g/ml) in a humidified atmosphere of 95% air and 5% CO_2_ at 37°C for 14 days. Cells were then fixed with glutaraldehyde (6.0% v/v) and stained with crystal violet (0.5% w/v) after washing with PBS. Colonies were counted manually under an inverted microscope (CKX53; Olympus Corporation, Tokyo, Japan) at 40× magnification.

### Wound healing assay

A total of 3 × 10^6^ cells were seeded in 6-well plates. When well confluence reached between 90% and 100%, a straight central linear wound was created in confluent cells using a 200-μl sterile pipette tip. Cells were subsequently rinsed twice with PBS to remove any debris prior to culture in serum-free growth medium. Wound healing was observed at different time points (0, 24, and 48 h), and the wound size was imaged using an inverted light microscope (CKX53; Olympus Corporation, 40× magnification). Finally, the distance between the two edges of the wound was measured using the Digimizer software system (version no. 5.3.4; MedCalc).

### Transwell invasion assay

The cell invasion ability of MKN-28/VCR cells was evaluated after 48 h using an 8-μm Matrigel invasion chamber (BD Bioscience), following the manufacturer’s instructions. The number of visible cells was counted in five random fields of view under a light microscope (CKX53; Olympus Corporation, 40× magnification).

### Identification of Siva-1-interacting proteins by yeast two-hybrid screening co-immunoprecipitation assay

Siva-1 was used as the bait, and a human cDNA library prepared from human gastric cancer was used as the prey. The AH109 yeast strain and Matchmaker two-hybrid system (Coolaber, Beijing, China) was used to perform yeast two-hybrid assay, according to the manufacturer’s instructions. To generate the bait plasmid, truncated deletion mutants of the Siva-1 gene was amplified using PCR and cloned into the pGBKT7 vector according to the PEG/LiAc method. A human cDNA library prepared from human gastric cancer was used as the prey and inserted into a pGADT7 prey vector. The yeast harboring pGBKT7 vector was transformed with a suitable cDNA library following the steps given in the instructions. To identify colonies positive for the bait, β-galactosidase assay was performed, and the identity of the prey was determined by PCR.

For the co-immunoprecipitation (co-IP) assay, total protein of cultured cells was lysed. We determined the protein concentration with BCA and incubated with 50 µl protein-A Sepharose beads (Biovision Inc., USA), and anti-Siva-1, anti-PCBP1, or anti-UXT antibodies, respectively as indicated, at 4°C overnight with gentle mixing. Anti-IgG was set as a control. Then, the immunoprecipitates were washed and denaturized for western blotting.

### Semiquantitative RT-PCR

Total RNA was extracted from MKN-28/VCR-shRNA-Siva-1, MKN-28/VCR-shRNA-NC, and MKN-28/VCR cells using TRIzol reagent (Invitrogen). All gene segments were amplified and verified using semiquantitative RT-PCR. cDNAs were reverse-transcribed from 2 μg total RNA. All PCR primer sequences used in this study, including those for Siva-1, PCBP1, Akt, NF-κB, MDR1, and MRP1, are shown in Table 1. The amplified products were separated by electrophoresis on ethidium bromide-stained 1.2% agarose gels. Band intensity was measured using ImageJ in three independent experiments. The values presented in the graph are average values of the three independent experiments. The ratio of target-to-control PCR products was determined by dividing the densitometric volume of the target band by that of the GAPDH band.

**Table 1:** Sequences of primers used for semiquantitative real-time polymerase chain reaction

Gene	Primer	Base sequence	PCR product (bp)
Siva-1	Forward	5′-TGTACCCTGTGTGGCCTCGT-3′	550
	Reverse	5′-AGCCAGCCTCAGGTCTCGAA-3′	
PCBP1	Forward	5′-CATCCGCTAAGAATTTAAAAAT-3′	1000
	Reverse	5′-AAGACAGCAATTCCCAGC-3′	
Akt	Forward	5′-GCTGGACGATAGCTTGGA-3′	383
	Reverse	5′-GATGACAGATAGCTGGTG-3′	
NF-κB	Forward	5′-TCCACTGTCTGCCTCTCTCGTC-3′	196
	Reverse	5′-GCCTTCAATAGGTCCTTCCTGC-3′	
MDR1	Forward	5′-GCTCCTGACTATGCCAAAGC-3′	201
	Reverse	5′-CTTCACCTCCAGGCTCAGTC-3′	
MRP1	Forward	5′-CTGTTTTGTTTTCGGGTTCC-3′	287
	Reverse	5′-GATGGTGGACTGGATGAGGT-3′	
GAPDH	Forward	5′-ACCACAGTCCATGCCATCAC-3′	450
	Reverse	5′-TCACCACCCTGTTGCTGTA-3′	

### Western blotting

Cytoplasmic proteins and nuclear proteins were extracted using a cell lysate extraction kit (Beijing Solarbio Science & Technology Co., Ltd., China) or a nuclear extraction kit (EpiGentek Group, Inc., Farmingdale, NY, USA), following the manufacturer’s protocol. The concentration of extracted protein was measured using a bicinchoninic acid protein assay kit (Thermo Fisher Scientific). equal amounts of proteins were loaded and separated via 12% SDS-PAGE, transferred to PVDF membranes, and blocked with 5% skimmed milk at 37°C for 1 h. Membranes were subsequently incubated with dilutions of primary antibody (anti-Siva-1: 1:100, anti-PCBP1: 1:1000, anti-Akt: 1:1000, anti-NF-κB: 1:1500, anti-MDR1: 1:1500, anti-MRP1: 1:1000, anti-Lamin: 1:1000, and anti-GAPDH: 1:1000), overnight at 4°C. The membranes were washed using TBS with 0.1% [v/v] Tween-20 (Thermo Fisher Scientific) and incubated with an HRP-conjugated goat anti-rabbit IgG H&L secondary antibody (1:1000) at 37°C for 1 h, according to the manufacturer’s recommendations. The western blot film was scanned, and net intensities of the bands were quantified using Image-QuanT software (Molecular Dynamics, Sunnyvale, CA, USA). GAPDH and Lamin B1 served as loading controls.

### Effect of Siva-1 silencing on reversing MDR in human gastric cancer MKN-28/VCR cells in vivo

When MKN-28/VCR cells were cultured to 90% cell confluency, cells were harvested using 0.25% trypsin and suspended in Matrigel (BD Bioscience). Five-week-old male BALB/c nude mice were subcutaneously inoculated in the flank with 2 × 10^6^ cells in 100 μL PBS; the resulting tumor was denoted an MKN-28/VCR tumor. When the resulting tumor measured 5 mm in diameter, animals were randomly divided into the following three groups (10 mice per group): MKN-28/VCR-shRNA-Siva-1, MKN-28/VCR-shRNA-NC, and MKN-28/VCR groups. The animals were respectively administered an intra-tumoral injection of 100 μL Lv-Siva-1-RNAi or LV-NC every 2 days. The viral titer was 5 × 10^8^ TU/ml medium, and an equal volume of PBS was used as a blank control. VCR was administered via intraperitoneal injection at a dose of 25 mg/kg every 2 days. Tumor volumes were monitored and analyzed every 2 days using a caliper. The tumor volume (TV) was calculated with the formula: TV = W^2^ × L/2, where W is the width and L is the length of the tumor. The relative tumor volume (RTV) was calculated with the formula: RTV = Vt/V0, where V0 is the TV at the day when chemotherapy was were given, and Vt is the TV of subsequent measurement. The mice were killed at 21 days after tumor injection, using 40 mg/kg of pentobarbital sodium anesthetic, and tumors were weighed and analyzed. Ethical approval for the animal study was obtained from the medical ethics committee of the People’s Hospital of Guangxi Zhuang Autonomous Region.

### Hematoxylin and eosin (HE) staining, immunohistochemistry, and TUNEL (terminal deoxynucleotidyl transferase dUTP nick-end labeling) assay

For HE staining, tumor tissues were fixed in 4% formaldehyde, dehydrated with gradient ethanol, and embedded in paraffin wax. Tissue sections were stained with HE after dewaxing and rehydration, according to a standard protocol. The rate of apoptosis was analyzed in a TUNEL assay with a detection kit (Boehringer Mannheim, Germany), as per the manufacturer. Cells were randomly counted with a light microscope (IX70; Olympus Corporation) in three independent experiments. The apoptotic index was calculated as apoptotic index = number of apoptotic cells/total number of cells. Immunohistochemistry was performed on formalin-fixed, paraffin-embedded serial sections (4 μm thick) of gastric cancer tissues using anti-Siva-1 antibody (1:2000). The immunostaining score was estimated based on the positive cell percentage and staining intensity of stained tumor cells by three independent pathologists. The percentage of positively stained cells was graded 0 (<5%), 1 (5%–25%), 2 (25%–50%), 3 (50%–75%), or 4 (>75%). Immunostaining intensity was rated as 0 (negative), 1 (weakly stained), 2 (moderately stained), or 3 (heavily stained). A positive immunoreaction (+) was when the product of multiplication between staining intensity and the percentage of positive cells was >5.

### Statistical analysis

Data are expressed as mean ± standard deviation. Statistical significance was determined using the χ^2^ test, Student *t*-test, or one-way analysis of variance. Statistical analyses were carried out using SPSS 13.0 (SPSS, Inc., Chicago, IL, USA). We set *p* < 0.05 to indicate a statistically significant difference.

## Results

### Siva-1 knockdown decreases the pump rate of doxorubicin

**Figure 1:**
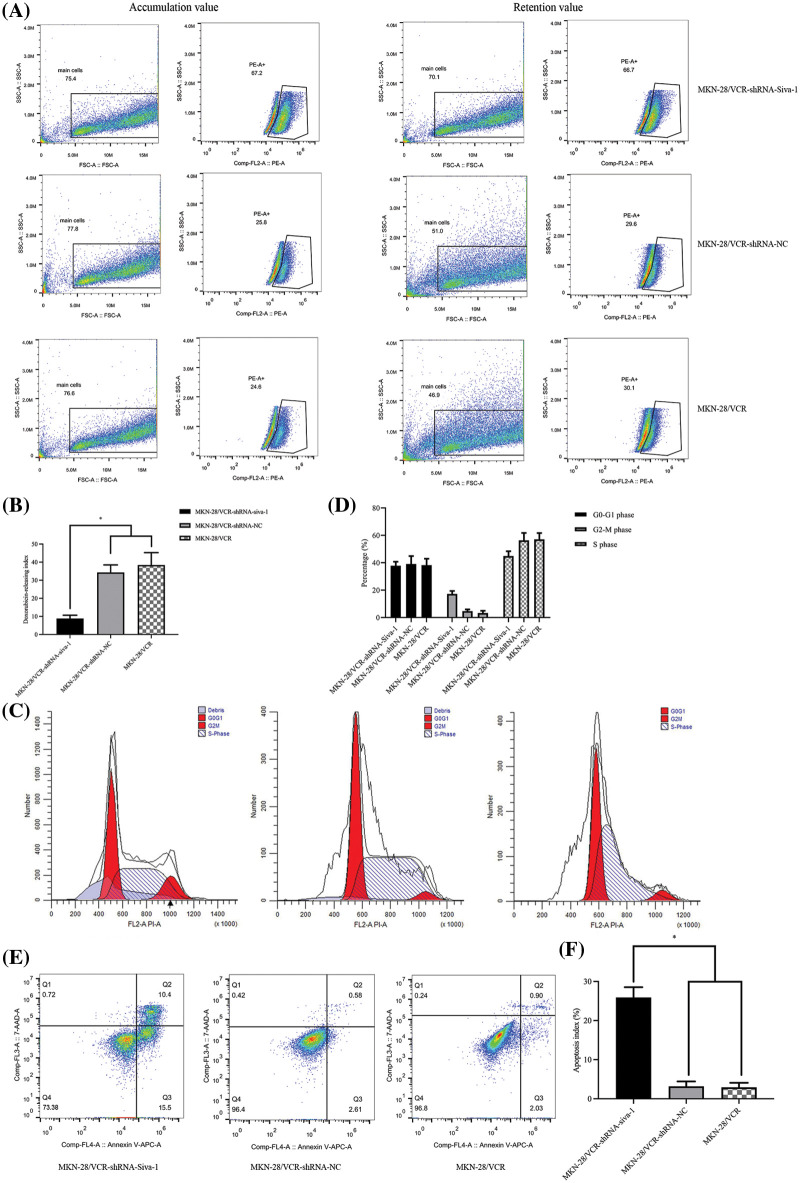
The effect on cell pump rate of doxorubicin, cell cycle, and apoptotic rate in MKN-28/VCR after Siva-1 was inhibited. (A, B) Pump rate of doxorubicin in MKN-28/VCR after Siva-1 was inhibited, analyzed by flow cytometry. (C, D) Cell cycle in MKN-28/VCR cells after Siva-1 was inhibited, analyzed by flow cytometry. (E, F) Apoptotic rate in MKN-28/VCR cells, analyzed by flow cytometry (*p < 0.05).

Decreasing the active pump-out ability of anti-tumor drugs and increasing the concentration of the drug in cells are known to be essential in antineoplastic combined chemotherapy. Intracellular drug accumulation and retention were evaluated using doxorubicin as a probe in gastric cancer cells. The pump rates of doxorubicin in MKN-28/VCR-shRNA-Siva-1 cells (7.03 ± 0.93%) were significantly lower than those in MKN-28/VCR-shRNA-NC cells (34.45 ± 8.31%) and MKN-28/VCR cells (38.78 ± 6.33%) (*p* < 0.05) (Figs. 1A and 1B).

### Siva-1 knockdown promotes cellular apoptosis and cell cycle arrest

As illustrated in Figs. 1C and 1D, MKN-28/VCR-shRNA-Siva-1 cells showed significantly different cell cycle distribution of S phase and G2/M. Siva-1 knockdown reduced the proportion of S phase in MKN-28/VCR cells, but the proportion of G2/M phase increased. The proportion of cells in S phase in MKN-28/VCR-shRNA-Siva-1 cells (44.91 ± 3.52%) was lower than those in MKN-28/VCR-shRNA-NC cells (57.58 ± 5.27%) and KN-28/VCR cells (57.08 ± 4.63%) (*p* < 0.05), whereas the proportion of cells in G2/M phase in MKN-28/VCR-shRNA-Siva-1 cells (17.26 ± 2.16%) was higher than those in MKN-28/VCR-shRNA-NC cells (4.68 ± 1.27%) and in MKN-28/VCR cells (3.29 ± 1.68%) (*p* < 0.05). Moreover, there was no significant difference in the cell cycle distribution of G0/G1. We found that the apoptosis rate was significantly enhanced after Siva-1 knockdown in MKN-28/VCR cells (25.91 ± 2.62%) (*p* < 0.05). Nevertheless, there was no difference between MKN-28/VCR-shRNA-NC cells (3.19 ± 1.25%) and MKN-28/VCR cells (2.93 ± 1.16%) (Figs. 1E and 1F).

### Siva-1 knockdown decreases proliferation of MKN-28/VCR cells

**Figure 2:**
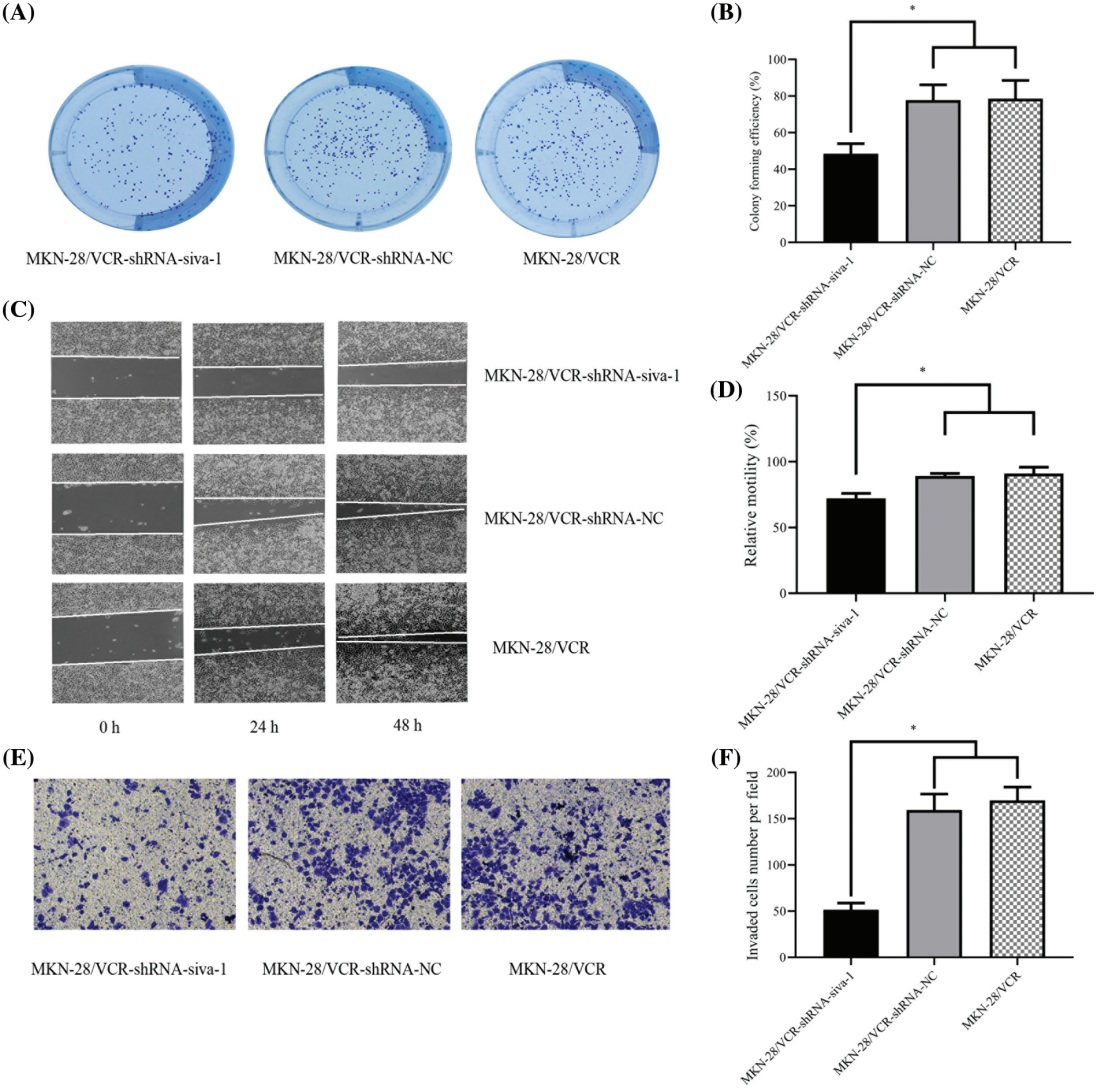
The effect on cell colony forming efficiency, the migratory rate, and invasion rate in MKN-28/VCR after Siva-1 was inhibited. (A, B) Proliferation rate of MKN-28/VCR after Siva-1 was inhibited, analyzed by cell colony assay. (C, D) Migratory rate of MKN-28/VCR after Siva-1 was inhibited, analyzed in wound healing assay. (E, F) Invasion rate of MKN-28/VCR after Siva-1 was inhibited, analyzed by transwell assay (*p < 0.05).

The results of the clone formation experiment showed that the number of cells in the MKN-28/VCR-shRNA-Siva-1 group (48.56 ± 5.38%) was significantly lower than those in the MKN-28/VCR-shRNA-NC group (77.81 ± 8.29%) and KN-28/VCR group (78.64 ± 9.86%) (*p* < 0.05) (Figs. 2A and 2B).

### Siva-1 knockdown restricts migration and invasion of MKN-28/VCR cells

To investigate the effect of Siva-1 knockdown on MKN-28/VCR cell migration and invasion, we performed wound healing and transwell assays, as described previously. We found that Siva-1 depletion markedly decreased the migration and invasion of MKN-28/VCR cells. The results of wound healing assays indicated that after culturing cells for 24 and 48 h, the relative mobility in MKN-28/VCR-shRNA-Siva-1 cells (72.21 ± 3.72%) was lower than that in the MKN-28/VCR-shRNA-NC group (89.15 ± 1.92%) and MKN-28/VCR group (91.07 ± 4.69%) (*p* < 0.05) (Figs. 2C and 2D). The results of transwell assays also indicated that, compared with the MKN-28/VCR-shRNA-NC (107.42 ± 9.29) and MKN-28/VCR (118.39 ± 10.24) groups, cells with Siva-1 depletion demonstrated markedly decreased invasion (66.72 ± 3.31) (*p* < 0.05) (Figs. 2E and 2F).

### Yeast two-hybrid screening of Siva-1

**Figure 3:**
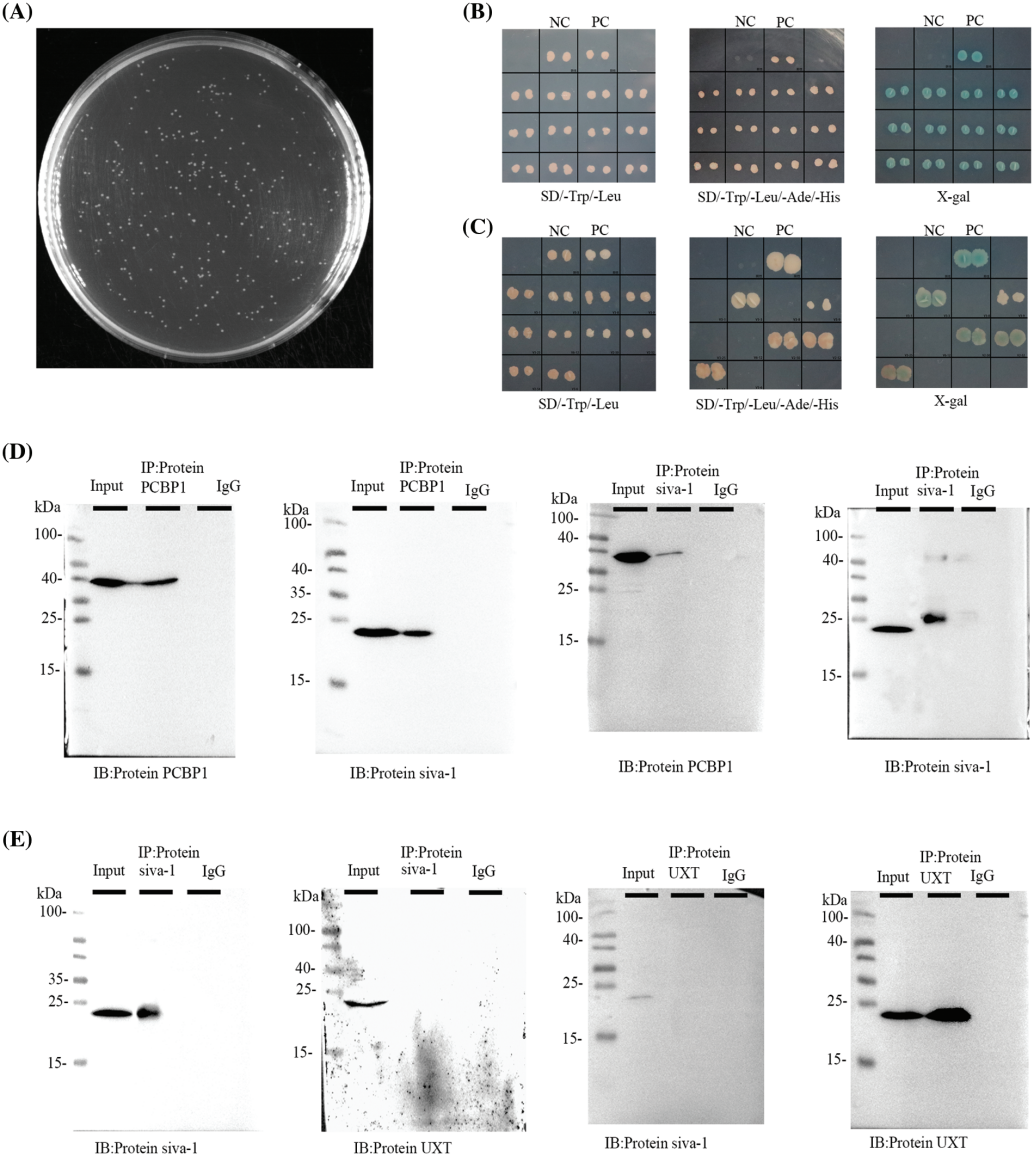
Siva-1 interacted with PCBP1. (A) Library quality test showed that the homogenized secondary yeast two-hybrid cDNA library capacity was 9.56 × 10^6^ CFU. (B, C) Yeast two-hybrid assays were performed to search for proteins that interacted with Siva-1. The bait vector with truncated Siva-1 gene was constructed for screening a human gastric cancer yeast two-hybrid cDNA library, and PCBP1 and UXT were identified. Interactions were confirmed based on co-transformants that can grow on histidine-deficient (-His) plates and exhibit β-galactosidase activity. (D) co-IP of Siva-1 with the PCBP1 protein. (E) co-IP to validated Siva-1 and UXT protein interaction in 293 T cells (*p < 0.05; NC: negative control; PC: positive control; IP: immunoprecipitation; IB: immunoblotting; SD: synthetic dropout medium).

To identify novel partners of Siva-1, we first performed yeast two-hybrid assay to find candidate proteins of this signaling pathway. cDNA and Siva-1 were cloned into plasmid pGBKT7, and both constructs were then co-transfected into yeast as bait protein to capture prey proteins (Fig. 3A). Three reporter genes induced by Gal4 (His3, Ade2, lacZ) were used for assessing protein–protein interactions. Yeast transformants were selected in minimal complete medium without Trp and Leu. Positive candidates were selected and grown in complete medium without Trp, Leu, Ade, and His. All positive clones were picked and were tested for β-galactosidase activity. and their DNA was extracted and sequenced. Finally, PCBP1 (LT741142.1) and UXT (NM-004182) were identified as candidate factors that potentially interact with Siva-1 (Figs. 3B and 3C).

### Siva-1 interacts with PCBP1

To confirm a direct physical association between Siva-1 and PCBP1, Siva-1 and UXT, we verified the interaction between them, respectively, by co-immunoprecipitation. We successfully observed the co-localization of Siva-1 and PCBP1 (Fig. 3D), demonstrating that these proteins do indeed interact. However, binding between Siva-1 and UXT could not be verified by co-immunoprecipitation as we did not find any co-localization of Siva-1 and UXT in MKN-28/VCR cells (Fig. 3E). Finally, we confirmed that Siva-1 interacted with PCBP1, which plays a pivotal role in regulating the multidrug-associated pathway.

### Siva-1 interacts with PCBP1 through inhibiting the Akt/NF-κB signaling pathway to reverse MDR

**Figure 4:**
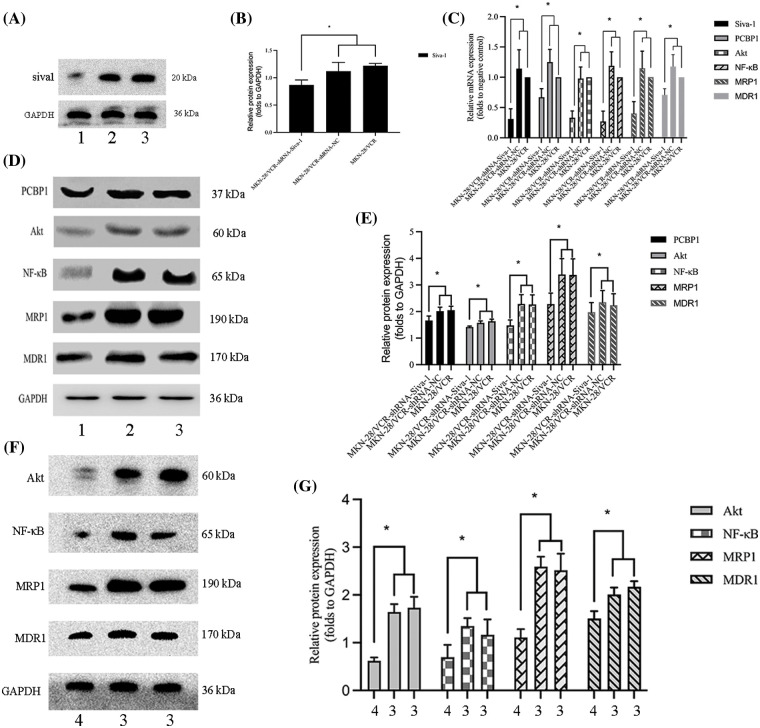
Siva-1 regulate PCBP1/Akt/NF-κB signaling pathway expression. (A, B) Relative expression levels of Siva-1 and its density quantification result. (C) Relative expression of Siva-1, PCBP1, Akt, NF-κB, MRP1, and MDR1 mRNA in MKN-28/VCR-shRNA-Siva-1 cells, MKN-28/VCR-shRNA-NC cells, and MKN-28/VCR cells. (D, E) Relative protein expression levels of PCBP1, Akt, NF-κB, MRP1, and MDR1 and their density quantification results. (F, G) Relative protein expression levels of Akt, NF-κB, MRP1, and MDR1 and their density quantification results (*p < 0.05; 1: MKN-28/VCR-shRNA-Siva-1; 2: MKN-28/VCR-shRNA-NC; 3: MKN-28/VCR; 4: MKN-28/VCR with Akt inhibitor).

To investigate the mechanism by which Siva-1 reverses MDR in human gastric cancer cells, we knocked down Siva-1 in MKN-28/VCR cells (Figs. 4A and 4B). Levels of some vital regulators (PCBP1, Akt, NF-κB) and several well-known MDR-associated proteins such as MDR1 and MRP1, were determined in semiquantitative RT-PCR and western blotting. The mRNA expression level of Siva-1, PCBP1, Akt, NF-κB, MDR1, and MRP1 in the MKN-28/VCR-shRNA-Siva-1 group was lower than those in the MKN-28/VCR-shRNA-NC group and MKN-28/VCR group (*p* < 0.05) (Table 2, Fig. 4C). Compared with the control group, the relative protein expression levels of Siva-1, PCBP1, Akt, NF-κB, MDR1, and MRP1 were significantly downregulated in the MKN-28/VCR-shRNA-Siva-1 group (*p* < 0.05) (Figs. 4D and 4E). In the experimental group, the Akt inhibitor (LY 294002) was added at the same as when MKN-28/VCR cells were inoculated with antibiotic-free medium. Compared with MKN-28/VCR cells incubated with medium alone, co-incubation with the Akt inhibitor (LY 294002) significantly inhibited NF-κB, MDR1, and MRP1 protein expression levels (Figs. 4F and 4G). These results indicate that Siva-1 inhibited the PCBP1/Akt/NF-κB pathway.

**Table 2:** mRNA expression levels of Siva-1, PCBP1, Akt, NF-κB, MDR1, and MRP1 in MKN-28/VCR cells after Siva-1 knockdown

Groups	Siva-1(2-ΔΔCt)	PCBP1(2-ΔΔCt)	Akt(2-ΔΔCt)	NF-κB(2-ΔΔCt)	MRP1(2-ΔΔCt)	MDR1(2-ΔΔCt)
MKN-28/VCR -shRNA-Siva-1	0.31 ± 0.17	0.67 ± 0.14	0.33 ± 0.11	0.27 ± 0.17	0.40 ± 0.19	0.71 ± 0.10
MKN-28/VCR -shRNA-NC	1.14 ± 0.31	1.25 ± 0.21	0.98 ± 0.19	1.19 ± 0.23	1.15 ± 0.28	1.30 ± 0.27
MKN-28/VCR	1.00	1.00	1.00	1.00	1.00	1.00

### Siva-1 knockdown reduces tumor burden and inhibits liver metastasis in vivo

**Figure 5:**
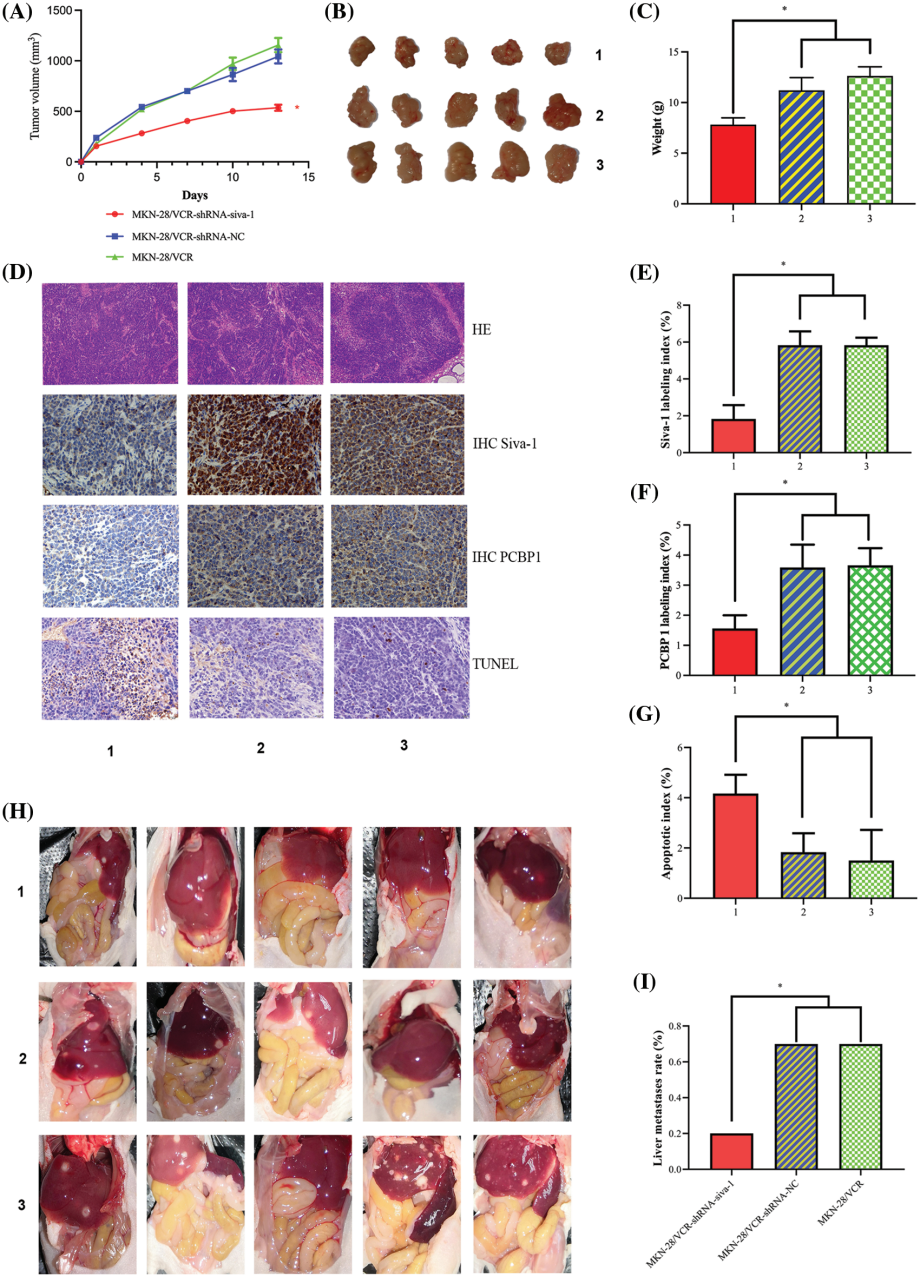
Inhibition of Siva-1 decreases tumor burden and liver metastases. (A) Volume development of subcutaneous tumor in mouse model. (B) Subcutaneous tumor tissue taken from nude mice when mice were killed on day 13. (C) Mouse body weight was measured when mice were killed on day 13. (D) Representative HE staining, immunohistochemical staining of Siva-1 and PCBP1, and TUNEL staining image in subcutaneous tumor tissue taken from nude mice in three different treatment groups. (E) Percentage of Siva-1-positive cells in three groups. (F) Percentage of PCBP1-positive cells in three groups. (G) Percentage of TUNEL-positive cells in three groups. (H) Representative images of liver metastases in nude mice inoculated with gastric cancer cells in different treatment groups. (I) Hepatic metastases rate of three different treatment groups (*p < 0.05; 1: MKN-28/VCR-shRNA-Siva-1; 2: MKN-28/VCR-shRNA-NC; 3: MKN-28/VCR; HE: hematoxylin and eosin; IHC: immunohistochemistry; TUNEL: terminal deoxynucleotidyl transferase dUTP nick end labeling).

To validate the biological function of Siva-1 in colorectal liver metastasis *in vivo*, we performed experimental metastasis assays in nude mice. The results showed that the growth of subcutaneously grafted tumors in the MKN-28/VCR-shRNA-Siva-1 group was clearly slower than that in the MKN-28/VCR-shRNA-NC group and MKN-28/VCR group (Figs. 5A and 5B). At the end of the experiment (Day 13), all tumors were isolated from the nude mice and weighed. The tumor weights in the Siva-1 knockdown group (7.13 ± 1.47 g) were significantly lower than those in the MKN-28/VCR-shRNA-NC group (10.31 ± 2.01 g) and MKN-28/VCR group (11.14 ± 1.72 g) (*p* < 0.05) (Fig. 5C), indicating better health. We then observed the expression of Siva-1 and PCBP1 in xenograft tumors using immunohistochemical analysis. We found that Siva-1 expression in the MKN-28/VCR-shRNA-Siva-1 group was predominantly lower than that in the MKN-28/VCR-shRNA-NC group and MKN-28/VCR group (Figs. 5D and 5E), meaning that Siva-1 was knocked down in MKN-28/VCR-shRNA-Siva-1 xenograft tumors. We also found that PCBP1 expression in the MKN-28/VCR-shRNA-Siva-1 group was significantly lower than that in the MKN-28/VCR-shRNA-NC group and MKN-28/VCR group (Figs. 5D and 5F) The mean percent of TUNEL-positive tumor cells was significantly higher in grafted tumors from the MKN-28/VCR-shRNA-Siva-1 group than in those from mice in the MKN-28/VCR-shRNA-NC and MKN-28/VCR (*p* < 0.05) groups (Figs. 5D and 5G), which revealed a significantly higher apoptosis rate in the Siva-1 knockdown group. We then examined the livers and found considerably more metastatic foci on their surface in mice from the MKN-28/VCR-shRNA-NC (70%) and MKN-28/VCR (70%) groups than in mice from the MKN-28/VCR-shRNA-Siva-1 group (20%) (*p* < 0.05) (Figs. 5H and 5I), indicating that Siva-1 knockdown significantly reduced the number of liver metastases.

## Discussion

Despite significant developments in methods of cancer treatment during the past decades, chemotherapy remains the main treatment for cancer. However, during chemotherapy, cancer cells evolve and can acquire MDR, which significantly limits the efficacy of cancer treatment and impacts patient survival and quality of life. It is therefore urgent to design chemotherapeutics that can evade or reverse MDR. Studies have analyzed MDR1 and MRP1 gene regulation by over-expressing Siva-1 and subsequently upregulating NF-κB in gastric cancer cell lines [5]. The aim of the present study was to further clarify the influences of Siva-1 on the mechanisms of cellular resistance to chemotherapy as well as the mechanisms of action in novel potential anticancer agents, which have been designed to overcome the resistance mechanisms.

Our previous study [5] indicated that Siva-1 overexpression inhibited apoptosis and enhanced MDR in a VCR-resistant gastric cancer cell line, and we also revealed that Siva-1 increased the colony formation and invasion of cells. Overexpression of Siva-1 in VCR-resistant cell lines decreased the sensitivity of cells toward VCR by enhancing the activity of NF-κB, thereby increasing the expression of MDR1 and MRP1 to enhance chemoresistance. To confirm the correlation between Siva-1 and MDR in gastric cancer cells, we inhibited the expression of Siva-1 and studied the phenomenon of MDR reversal. The results indicated that inhibition of Siva-1 expression prevented drug efflux in gastric cancer cells, increased the sensitivity of cells to the chemotherapeutic drug VCR, decreased the number and size of cancer cell colonies, and increased the cell apoptosis rate. We also found that the number of cells in G1 phase markedly increased, whereas those in S phase decreased in Siva-1-downregulated cells. Our study revealed that inhibition of Siva-1 expression in MKN-28/VCR cells significantly weakened wound healing ability and decreased invasion ability. It is therefore crucial to elaborate the role played by Siva-1 in MDR and the mechanisms of cellular resistance to chemotherapy.

We performed yeast two-hybrid screening and identified PCBP1 as a Siva-1 interaction partner and revealed that their interaction is functionally critical to the NF-κB signaling pathway. Thus, these were chosen for subsequent experiments to further explore the function of Siva-1 in reversing MDR in gastric cancer. We detected expression levels of PCBP1, Akt, NF-κB, MDR1, and MRP1 in semiquantitative RT-PCR and western blot analysis. The results showed that the expression of PCBP1, Akt, NF-κB, MDR1, and MRP1 were suppressed when Siva-1 was factitiously silenced.

PCBP1 is a multifunctional adaptor protein initially identified as an RNA-binding protein. PCBP1 expression was significantly higher in tumor samples from oxaliplatin-refractory patients than in those from oxaliplatin-responsive patients. A past study showed that knockdown of PCBP1 sensitized human oxaliplatin-resistant colorectal cancer cells (HT-29/L-OHP) to oxaliplatin, whereas overexpression of PCBP1 increased oxaliplatin resistance in colorectal cancer cells [9]. Furthermore, knockdown of PCBP1 inhibits the activation of Akt in human colorectal cancer cells [10,11]. The crucial roles of PCBP1 have been revealed in transcriptional and translational events, influencing the expression of the PCBP1 downstream gene Akt [12,13]. Overexpressed PCBP1 significantly increased levels of Akt activation; In contrast, knockdown of PCBP1 downregulated and inhibited Akt activation. The activation of Akt signaling was linked to increased expression of PCBP1. Interestingly, Akt 2 was reported to be able to phosphate PCBP1 and regulate its activity, thus indicating a strong connection between PCBP1 and the Akt signaling pathway [10,14,15]. Together, these findings suggest that PCBP1 is mainly involved in the Akt signaling pathway. NF-κB is a downstream component of the Akt pathway and is activated via phosphorylation of IκB kinase (IKK), leading to IκB degradation. The Akt/NF-κB pathway has been reported to regulate the cisplatin sensitivity in lung adenocarcinoma cells [16], cisplatin sensitivity in human breast cancer cells [17], chemosensitivity to cisplatin in human NSCLS cells [18], cisplatin resistance in gastric cancer cells [19], and carboplatin sensitivity in prostate cancer [20]. Notably, the Akt and NF-κB signaling pathways are appealing for their crucial role in the pathogenesis and progression of chemical drug resistance [21]. For example, elevated levels of activated NF-κB are also associated with chemoresistance by regulating target genes, such as inhibitors of apoptosis [22,23]. NF-κB activation is one mechanisms contributing to tumor resistance to chemotherapeutic agents. Interestingly, the chemotherapy-induced EMT phenotype is another mechanism for drug resistance. Current strategies to reverse EMT to MET are a promising approach to overcome drug resistance. Furthermore, upon NF-κB activation, the enhanced expression of antiapoptotic genes, such as c-IAP, Survivin, Bcl-xL, MDR1, and MRP1 induced by NF-κB also contribute to tumor resistance. Upon NF-κB activation, two main mechanisms account for resistance to chemotherapy agents. First, since the location −444 to −435 of human SNAIL promoter has an NF-κB binding site, the promoter of SNAIL is upregulated when NF-κB is activated, resulting in the decrease of E-cadherin and acquisition of the EMT phenotype. Second, NF-κB activation attenuates apoptosis by regulating antiapoptotic genes, resulting in the survival of cancer cells13. Thus, the Akt/NF-κB pathway induces tumor cell resistance to chemotherapy agents by inducing the EMT phenotype and enhancing antiapoptotic gene expression. Therefore, inhibition of PCBP1/Akt/NF-κB has a therapeutic role in overcoming chemoresistance [24,25].

In the current study, we hypothesized that suppressed NF-κB levels mediated by Siva-1 could decrease gastric cancer cell metastasis and reverse MDR. The two-hybrid yeast study revealed that downregulated Siva-1 attenuates the expression of PCBP1, indicating that Siva-1 directly interacts with PCBP1. PCBP1 plays a pivotal role in regulating MDR1 and MRP 1 by involvement in the Akt/NF-κB pathways. Given the crucial role of Siva-1 in the regulation of MDR and gastric cancer cell metastasis, Siva-1 could serve as a novel potential anticancer agent designed to overcome chemotherapeutic resistance mechanisms.

## Data Availability

All data and materials are available from Dr. Xiaotong Wang.
